# Association Between Creatinine and Lung Cancer Risk in Men Smokers: A Comparative Analysis with Antioxidant Biomarkers from the KCPS-II Cohort

**DOI:** 10.3390/antiox14050584

**Published:** 2025-05-12

**Authors:** Jong-Won Shin, Thien-Minh Nguyen, Sun-Ha Jee

**Affiliations:** 1 Department of Laboratory Medicine, Asan Medical Center, University of Ulsan College of Medicine, Seoul 05505, Republic of Korea; jongwon_shin@amc.seoul.kr; 2 Department of Epidemiology and Health Promotion, Institute for Health Promotion, School of Public Health, Yonsei University, 50-1 Yonsei-ro, Seodaemun-gu, Seoul 03772, Republic of Korea; nguyenminh2301@gmail.com; 3 Department of Epidemiology, Faculty of Public Health, University of Medicine and Pharmacy, Ho Chi Minh City 17000, Vietnam

**Keywords:** creatinine, antioxidants, lung neoplasms, smoking, biomarkers

## Abstract

Bilirubin, albumin, and uric acid are established endogenous antioxidant biomarkers, whereas the antioxidant role of creatinine has not yet been fully clarified. As a byproduct of creatine metabolism, creatinine may reflect underlying metabolic activity and redox balance, particularly under conditions of oxidative stress such as cigarette smoking. This study aimed to evaluate the associations between serum creatinine and other antioxidant biomarkers and lung cancer risk, stratified by smoking status. We analyzed 83,371 cancer-free men from the Korean Cancer Prevention Study II (KCPS II) cohort. During a mean follow-up of 13.5 years, 533 incident lung cancer cases were identified. Serum creatinine, total bilirubin, albumin, and uric acid were measured. Smoking status classified participants as never-, former, and ever-smokers, with ever-smokers including both current and former smokers. Cox proportional hazards regression models estimated hazard ratios (HRs) and 95% confidence intervals (CIs), stratified by smoking status. Biomarkers were also analyzed by quartiles and linear trends. A single standard deviation increase in serum creatinine was significantly and inversely associated with lung cancer risk among former smokers (HR: 0.774, 95% CI: 0.620 to 0.967) and ever-smokers (HR: 0.823, 95% CI: 0.716 to 0.945). Total bilirubin also showed significant inverse associations in former smokers (HR: 0.826, 95% CI: 0.705 to 0.967) and ever-smokers (HR: 0.785, 95% CI: 0.708 to 0.870). Albumin was inversely associated only with ever-smokers (HR: 0.878, 95% CI: 0.807 to 0.955), while uric acid showed inverse associations with both former smokers (HR: 0.832, 95% CI: 0.699 to 0.989) and ever-smokers (HR: 0.847, 95% CI: 0.760 to 0.944). None of the biomarkers showed significant associations among never-smokers. Serum creatinine and other endogenous antioxidant biomarkers were inversely associated with lung cancer risk, particularly in individuals with a history of smoking exposure.

## 1. Introduction

Lung cancer is one of the leading causes of cancer-related mortality worldwide [1,2], and the incidence and mortality rates are substantially higher in men than in women. This sex disparity is believed to result not only from differences in smoking prevalence but also from biological and pathophysiological distinctions in disease mechanisms [3,4,5]. The development of lung cancer is known to be driven by both genetic factors and environmental exposures, with smoking-induced oxidative stress playing a central role. Smoking increases the production of reactive oxygen species (ROS) and reactive nitrogen species (RNS), which contribute to lung carcinogenesis through DNA damage, chronic inflammation, apoptosis, and immune suppression [6]. In contrast, lung cancer in never-smokers is thought to be driven by a combination of different mechanisms, including exposure to environmental carcinogens such as radon and indoor air pollution, genetic mutations, and hormonal factors. These findings suggest that the etiological pathways of lung cancer may differ based on smoking status [7,8,9,10,11]. Given the oxidative stress-related mechanisms in lung cancer development, increasing attention has been paid to endogenous antioxidant biomarkers that may help prevent or predict lung cancer risk at an early stage. Total bilirubin, albumin, and uric acid are representative endogenous antioxidants, and their protective associations have been reported in various oxidative stress-related cancers [12,13,14,15,16]. Their relevance in lung cancer has also been increasingly recognized [17,18,19]. However, large-scale prospective studies stratifying participants by smoking status to examine the associations between these biomarkers and lung cancer risk remain limited. In addition to these established antioxidant markers, this study focused on the potential antioxidant relevance of serum creatinine, which has traditionally been considered a marker of kidney function or muscle mass. Although serum creatinine has not been directly linked to antioxidant metabolism from a physiological standpoint, previous studies have used indices such as the uric acid/creatinine ratio (UA/Cr) and creatinine-to-cystatin C ratio (Cr/CysC) as surrogate indicators of muscle mass or metabolic state [20,21]. Notably, these indices have shown significant associations with lung function, providing indirect support for the hypothesis that creatinine may reflect oxidative or antioxidant status. Therefore, this study aimed to evaluate the associations between serum creatinine and other endogenous antioxidant biomarkers with the risk of lung cancer, stratified by smoking status, using data from the large-scale, prospective Korean Cancer Prevention Study-II (KCPS-II) cohort. In doing so, we also sought to explore the potential role of creatinine as a physiological marker reflecting antioxidant status.

## 2. Materials and Methods

### 2.1. Study Population

This study was based on data from the Korean Cancer Prevention Study II (KCPS-II), a large-scale prospective cohort comprising 153,971 adults aged 20 to 84 years (mean age: 42, SD: 11) who underwent health examinations at 18 centers across South Korea between 2004 and 2013. All participants provided written informed consent for the use of their health screening and questionnaire data for research purposes [22]. Individuals with a prior history of cancer at baseline were excluded, as well as those with missing data on key variables, including smoking status, alcohol consumption, and serum levels of bilirubin, albumin, uric acid, and creatinine. After applying these exclusion criteria, a total of 133,630 participants were included in the analysis. The primary objective of this study was to evaluate the associations between endogenous antioxidant biomarkers, including creatinine, bilirubin, albumin, and uric acid, and the risk of lung cancer in men, stratified by smoking status. Therefore, the final analysis was restricted to 83,371 male participants. This study was approved by the Institutional Review Board of Severance Hospital, Yonsei University Health System (approval number: 4-2011-0277).

### 2.2. Cancer Case Ascertainment

The occurrence of cancer among study participants was verified annually with nearly 100% accuracy by linking data from the National Cancer Center (NCC) Registry using resident registration numbers [22]. In Korea, under the Cancer Control Act, all hospitals are mandated to report cancer diagnoses to the NCC. Cancer cases were classified according to the 10th revision of the International Classification of Diseases (ICD-10). The average follow-up duration for the cohort was 13.5 years. Specifically, for lung cancer (C34), the median follow-up time among men was 13.7 years (IQR: 13.3–14.4) for never-smokers, 14.2 years (IQR: 13.4–14.7) for former smokers, and 14.1 years (IQR: 13.4–14.6) for ever-smokers. A total of 553 incident cases of lung cancer were identified among men during the follow-up period.

### 2.3. Statistical Analysis

Baseline characteristics of the study population were summarized using descriptive statistics according to smoking status (never-smokers, former smokers, and ever-smokers). The four endogenous antioxidant biomarkers of interest included serum creatinine, total bilirubin, albumin, and uric acid. Each biomarker was standardized by its respective standard deviation (SD). Cox proportional hazards models were used to examine the association between a 1-SD increase in each biomarker and the risk of lung cancer. All models were adjusted for potential confounding variables, including age, alcohol consumption, body mass index (BMI), serum glutamate oxaloacetate transaminase (GOT), and gamma-glutamyl transferase (GGT). In addition, each biomarker was categorized into quartiles. Hazard ratios (HRs) and 95% confidence intervals (CIs) were estimated using the lowest quartile as the reference category. Linear trends across quartiles were assessed by assigning the median value of each quartile and modeling it as a continuous variable. Smoking status categorized participants as never-smokers, former smokers, and ever-smokers, with the ever-smoker group including both current and former smokers. Information on smoking habits was collected through a baseline self-reported questionnaire, based on the cohort profile of the Korean Cancer Prevention Study-II (KCPS-II) Biobank [22]. Serum levels of creatinine, total bilirubin, albumin, and uric acid were measured in the clinical laboratories of the hospitals where participants were enrolled. All laboratories adhered to both internal and external quality control procedures in accordance with the standards of the Korean Association of Laboratory Quality Control. The inter-laboratory correlation coefficients for these measurements ranged from 0.96 to 0.99, ensuring high accuracy and consistency across sites [22]. For serum creatinine, a sensitivity analysis was conducted by removing BMI from the adjustment model to assess the robustness of the observed association. An additional multivariable-adjusted analysis was also performed in the overall male population to further evaluate the association between creatinine and lung cancer risk. To minimize potential reverse causality due to latent cancer, we conducted a separate sensitivity analysis excluding lung cancer cases diagnosed within the first three years of follow-up. All statistical analyses were performed using SAS version 9.4 (SAS Institute Inc., Cary, NC, USA). Person-years were calculated from the date of baseline examination to the date of lung cancer diagnosis, death, or the end of follow-up, whichever occurred first.

## 3. Results

### Characteristics of the Study Population

As shown in Table 1, the study population included 133,596 participants, of whom 83,371 were men. The mean age was 40.9 years (SD: 10.0) overall and 41.6 years (SD: 9.5) among men. The variables analyzed in this study included body mass index (BMI), serum creatinine, bilirubin (total, indirect, and direct), albumin, uric acid, platelet count, alcohol consumption, and smoking status. Smoking status categorized participants as never smokers, former smokers, and current smokers, while alcohol consumption was grouped into never drinkers, former drinkers, and current drinkers.

Smoking status in men was categorized into never-smokers, former smokers, and ever-smokers, the latter including both current and former smokers. This classification was consistently applied in all analyses.

**Figure 1:**
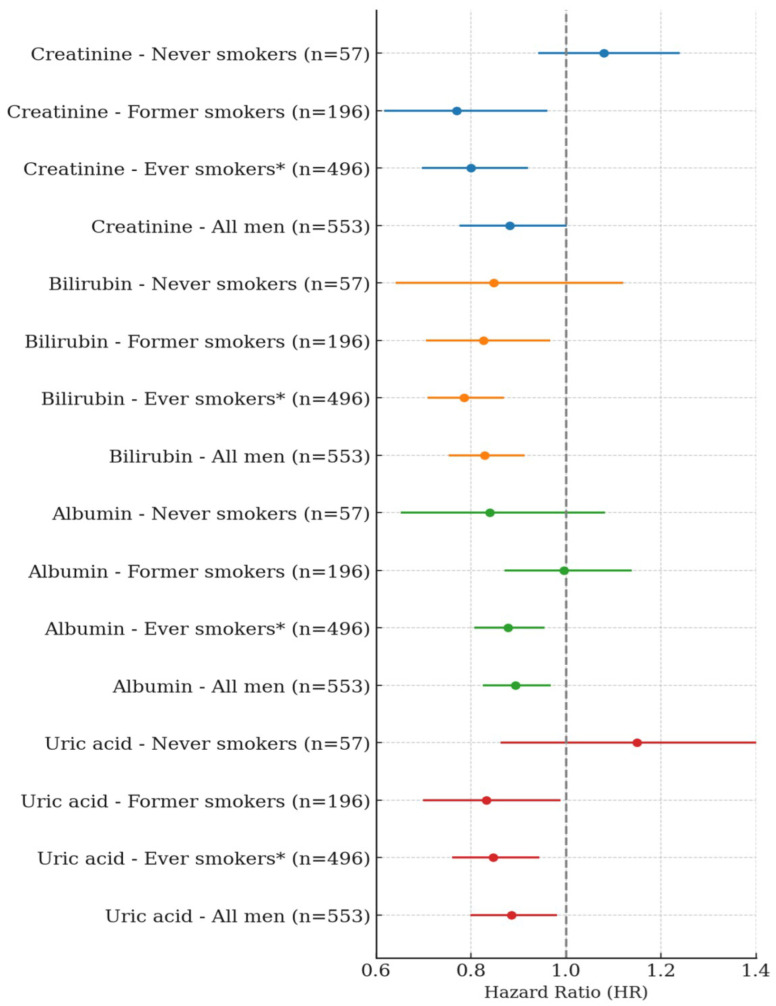
Summary of Lung Cancer Risk per 1-SD Increase in Serum Biomarkers by Smoking Status in Men. * Ever smokers were defined as current or former smokers. This figure provides a visual summary of the hazard ratios and 95% confidence intervals presented in Table 2 and Table 3.

In men, a single SD increase in serum creatinine was significantly and negatively associated with lung cancer risk among former smokers and ever-smokers. Specifically, a single SD increase in creatinine showed a negative association of 22.6% in former smokers (HR: 0.774, 95% CI: 0.620–0.967, *p*-value = 0.0239) and 17.7% in ever-smokers (HR: 0.823, 95% CI: 0.716–0.945, *p*-value = 0.0057). When BMI was excluded from the model, the negative associations were slightly stronger, with HRs of 0.770 in former smokers (95% CI: 0.617–0.961, *p*-value = 0.0209) and 0.800 in ever-smokers (95% CI: 0.697–0.920, *p*-value = 0.0017). Among never-smokers, no significant association was observed. In the overall male population, a single SD increase in creatinine showed a trend toward a negative association, which approached statistical significance in the BMI-excluded model (HR: 0.882, 95% CI: 0.776–1.002, *p*-value = 0.0541) (Table 2, Figure 1). A single SD increase in total bilirubin was significantly and negatively associated with lung cancer risk among former smokers and ever-smokers. Specifically, total bilirubin showed a 17.4% negative association in former smokers (HR: 0.826, 95% CI: 0.705–0.967, *p* = 0.0177) and a 21.5% negative association in ever-smokers (HR: 0.785, 95% CI: 0.708–0.870, *p* < 0.0001). In the overall male population, total bilirubin was also significantly and negatively associated with lung cancer risk (HR: 0.829, 95% CI: 0.753–0.913, *p* = 0.0001). No significant association was observed among never-smokers. A one SD increase in albumin was significantly and negatively associated with lung cancer risk only among ever-smokers (HR: 0.878, 95% CI: 0.807–0.955, *p* = 0.0024), and this association remained significant in the overall male population (HR: 0.893, 95% CI: 0.825–0.968, *p* = 0.0059). No significant associations were observed among never-smokers or former smokers. Similarly, a one SD increase in uric acid was negatively associated with lung cancer risk in both former smokers (HR: 0.832, 95% CI: 0.699–0.989, *p* = 0.0367) and ever-smokers (HR: 0.847, 95% CI: 0.760–0.944, *p* = 0.0027). This inverse association was also observed in the overall men population (HR: 0.885, 95% CI: 0.799–0.981, *p* = 0.0203). No significant association was found among never-smokers (Table 3, Figure 1).

**Figure 2:**
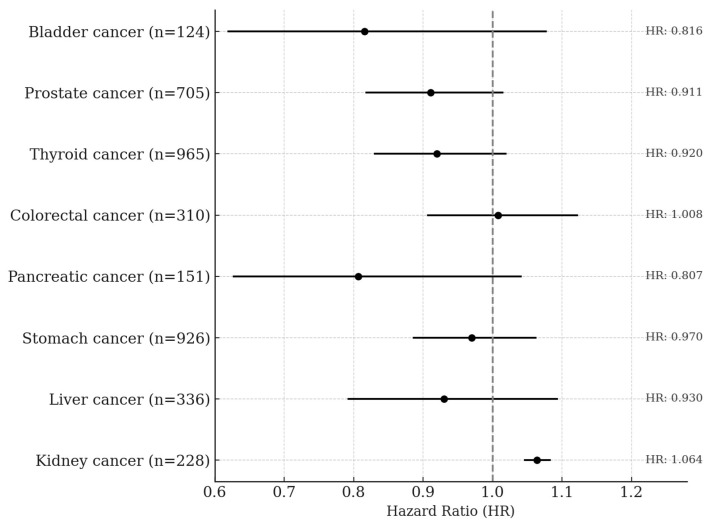
Site-Specific Cancer Risk per 1-SD Increase in Serum Creatinine Levels Among Men. The Cox proportional hazards model was adjusted for age, sex, smoking status, alcohol consumption, body mass index, GOT, and GGT.

In the quartile-based analysis, serum creatinine showed approximately a 39% negative association with lung cancer risk among former smokers, and negative associations of 31% and 24% were observed in the Q3 and Q4 quartiles, respectively, among ever-smokers. In the overall male population, the Q3 quartile also showed a 26% lower risk of lung cancer, and these associations became slightly stronger in the BMI-excluded model. A statistically significant trend across quartiles was observed among ever-smokers (*p* for trend = 0.0063) and in the BMI-excluded model (*p* for trend = 0.0019). No significant associations or trends were observed among never-smokers (Table 4). Total bilirubin showed negative associations with lung cancer risk of 28% in Q3 and 42% in Q4 among ever-smokers. In the overall male population, risk reductions of 24% and 31% were also observed in Q3 and Q4, respectively. Statistically significant trends were observed both among ever-smokers (*p* for trend < 0.0001) and in the overall male population (*p* for trend = 0.0009). No significant associations or trends were observed among never-smokers or former smokers. Albumin showed a significant negative association only among ever-smokers in the Q4 quartile, with a risk reduction of approximately 22%, and the trend across quartiles was also statistically significant (*p* for trend = 0.0183). No significant associations or trends were observed among never-smokers, former smokers, or in the overall men population. Uric acid showed a 36% negative association with lung cancer risk in the Q4 quartile among former smokers, and among ever-smokers, the Q3 and Q4 quartiles showed risk reductions of 35% and 28%, respectively. In the overall male population, the Q3 quartile also showed a 27% lower risk of lung cancer. Statistically significant trends were observed among former smokers (*p* for trend = 0.0244), ever-smokers (*p* for trend = 0.0016), and in the overall male population (*p* for trend = 0.0134). No significant associations or trends were found among never-smokers (Table 5). In men, a one standard deviation increase in serum creatinine was not significantly associated with the risk of any cancer type except kidney cancer. A significant positive association was observed for kidney cancer, with a hazard ratio of 1.064 (95% CI: 1.045–1.084, *p* < 0.0001) (Figure 2).

## 4. Discussion

This study, based on a large-scale prospective cohort, investigated the associations between serum creatinine and other endogenous antioxidant markers with lung cancer risk stratified by smoking status. Creatinine has traditionally been used as a metabolic byproduct reflecting kidney function or muscle mass, and most epidemiological studies have interpreted it within this metabolic context. However, recent biochemical studies have suggested that the creatine metabolic pathway may exert direct antioxidant effects through the scavenging of reactive oxygen species (ROS) [23,24]. As the final metabolic product of this pathway, creatinine may indirectly reflect the body’s antioxidant status, which formed the physiological rationale for the present study. GOT (AST) and GGT are not only indicators of liver function but are also closely associated with oxidative stress and antioxidant systems [25]. In particular, GGT is involved in glutathione (GSH) metabolism, and GSH is a potent endogenous antioxidant that plays a critical role in tumor progression and resistance to anticancer therapy. Therefore, in this study, we included GOT and GGT as covariates to adjust for potential influences of liver function and oxidative stress status when evaluating the association between serum creatinine and lung cancer risk. Creatinine is closely related to muscle mass, and body mass index (BMI) is also widely recognized as an indicator of body composition and metabolic state. In fact, previous studies have reported a strong correlation between BMI and serum creatinine levels [26,27,28], a relationship explained by physiological mechanisms rooted in muscle mass and body size. Based on this, we conducted a sensitivity analysis excluding BMI as a covariate to evaluate whether the observed association between creatinine and lung cancer risk might be influenced by BMI. The results showed that the inverse association between creatinine and lung cancer risk remained consistent even after BMI was excluded from the model, with the association slightly strengthened. These findings suggest that BMI may not simply act as a confounder but rather may represent a component of the physiological pathway through which creatinine influences lung cancer development. Alternatively, BMI adjustment may partially attenuate the biological signal of the antioxidant status reflected by creatinine. In this context, creatinine may function not merely as a marker of metabolism or body composition but as an independent biomarker reflecting antioxidant status in oxidative stress-related conditions such as smoking. Moreover, serum creatinine has recently been used as a surrogate marker for muscle mass, and a few studies have explored its association with cancer prognosis or mortality across various cancer types. Accordingly, our study also examined the associations between creatinine and the risk of major cancers other than lung cancer (Figure 2). The results showed no statistically significant associations for most cancer types including liver, gastric, colorectal, pancreatic, prostate, thyroid, and bladder cancers. A significant positive association was observed only for kidney cancer, which aligns with the well-established physiological relationship between creatinine and renal function. These findings support the idea that creatinine may reflect different physiological characteristics depending on the cancer type and underscore its potential role as an independent antioxidant biomarker, particularly in cancers like lung cancer where oxidative stress plays a central role in pathogenesis.

### 4.1. Endogenous Antioxidant Biomarkers

Bilirubin, albumin, and uric acid are well-established endogenous antioxidant substances. Among them, bilirubin has been particularly well documented to exert a protective role in various cancers associated with oxidative stress [12,13,14,15,16]. Bilirubin acts as a lipophilic antioxidant by suppressing lipid peroxidation in cell membranes and scavenging reactive oxygen species (ROS). Even after oxidation, it can be regenerated by biliverdin reductase, enabling a continuous antioxidant cycle, which underscores its physiological importance [29,30,31]. In addition to its association with lung cancer risk, a recent Mendelian randomization (MR) study suggested a causal inverse relationship, showing that genetically elevated bilirubin levels are associated with reduced lung cancer risk [17]. Albumin, the most abundant protein in plasma, inhibits ROS production by binding metal ions and suppressing the Fenton reaction. It also directly removes free radicals through its thiol group (–SH group) [32]. Furthermore, albumin plays anti-inflammatory roles, which may influence the pathophysiology of cancers associated with systemic inflammation. Uric acid is a potent water-soluble antioxidant capable of effectively neutralizing not only ROS such as superoxide and hydrogen peroxide but also reactive nitrogen species (RNS) like peroxynitrite. These actions help reduce oxidative DNA damage and contribute to membrane stabilization across various tissues, including the lungs. Moreover, recent studies have also proposed a potential causal association between uric acid and lung cancer [19]. In our study, when these antioxidant markers were analyzed together, serum creatinine showed an inverse association with lung cancer risk comparable to that of bilirubin, with the association being more pronounced among smokers. Albumin and uric acid also exhibited statistically significant inverse associations, although their magnitudes were relatively smaller compared to that of creatinine. These findings suggest that creatinine, beyond its conventional interpretation as a marker of muscle mass or kidney function, may act as a physiological indicator that directly or indirectly reflects antioxidant status in the context of oxidative stress. In cancers like lung cancer, where oxidative stress plays a central pathogenic role, creatinine may serve as an epidemiologically meaningful marker comparable to established endogenous antioxidants.

### 4.2. Creatine Metabolism, Antioxidant Activity, and the Role of Serum Creatinine

Serum creatinine has traditionally been regarded as a metabolic byproduct reflecting muscle mass or renal function, without a clearly defined biological role. However, as the end-product of creatine metabolism, it may indirectly reflect metabolic activity or redox homeostasis. Creatine is synthesized in the liver and kidneys and transported to skeletal muscle, where it is stored as phosphocreatine by binding with ATP. During energy-demanding states, creatine helps regenerate ATP and is spontaneously and non-enzymatically converted to creatinine at a steady rate of approximately 1.5–2% per day. Unlike creatine, which is primarily stored intracellularly and has low and variable serum levels, creatinine is consistently produced and stably measured in blood. These properties make serum creatinine a practical and reliable indicator of metabolic and physiological status. Furthermore, recent experimental studies have demonstrated that creatine itself has direct antioxidant properties, suggesting that its downstream metabolite, creatinine, may serve as an indirect biomarker of antioxidant capacity under certain physiological conditions [23,24]. In our analysis, serum creatinine showed a statistically significant inverse association with lung cancer risk, especially among smokers. This suggests that under oxidative stress conditions, such as those induced by smoking, the creatine–creatinine pathway may reflect endogenous antioxidant activity. This pattern is consistent with those observed for other well-established endogenous antioxidant biomarkers, including bilirubin, albumin, and uric acid. Additionally, as creatinine is filtered and excreted by the kidneys, it has a strong physiological association with renal function. In this study, a significant association was observed only for kidney cancer among major cancer types other than lung cancer, suggesting that the physiological significance of creatinine may vary depending on cancer type. In the case of kidney cancer, it may have acted as a marker reflecting impaired renal function. These findings are supported by prior studies. Decreased estimated glomerular filtration rate (eGFR) and elevated serum creatinine levels have been linked to increased risk of kidney cancer and overall cancer incidence [33]. Other studies have reported a positive association between serum creatinine and prostate cancer [34], as well as associations between albuminuria, impaired renal function, and multiple cancer types [35]. These findings collectively suggest that serum creatinine may reflect different physiological mechanisms depending on the type of cancer. For example, in lung cancer, where oxidative stress plays a key pathogenic role, creatinine may function as a surrogate marker of antioxidant status, whereas in kidney cancer, it may serve as an indicator of impaired renal function. Such dual roles support the potential of creatinine as an extended biomarker that goes beyond kidney function to encompass metabolic activity and redox balance, providing meaningful insights in epidemiological research.

In a study of patients with traumatic brain injuries, serum creatinine levels along with uric acid, bilirubin, and albumin showed significant changes during the acute phase, with sex-specific differences in antioxidant status [36]. Similarly, patients with myasthenia gravis were found to have lower levels of serum creatinine and other antioxidant markers compared to those in healthy controls, suggesting diminished antioxidant defense mechanisms [37]. These prior findings indicate that creatinine may act as a marker of endogenous antioxidant status across a range of disease contexts, consistent with our findings.

Although the exact biochemical mechanisms linking creatinine to antioxidant function have yet to be fully elucidated, the significant epidemiological associations observed in this study highlight the need for further mechanistic research. Future investigations incorporating the creatine-to-creatinine ratio, genetic factors related to creatine metabolism, and biomarkers of oxidative stress may provide more definitive insights into this pathway. Furthermore, given that serum creatinine is stable, cost-effective, and easily accessible, our findings suggest its potential utility as a screening biomarker or a component of lung cancer risk prediction models in both clinical and public health settings. Further studies are warranted to explore its broader applicability and clinical relevance.

This study has several limitations that warrant consideration. First, all antioxidant biomarkers, including serum creatinine, were measured only once at baseline health examinations, and thus, potential physiological fluctuations or long-term changes could not be captured. Second, although adjustments were made for major confounders such as age, alcohol consumption, BMI, GOT, and GGT, the possibility of residual confounding due to unmeasured variables cannot be completely ruled out. Third, while the analysis was stratified by smoking status, detailed indicators of smoking exposure, such as cumulative pack-years or secondhand smoke exposure, were not available, limiting the depth of the analysis. Fourth, histological subtypes of lung cancer (e.g., adenocarcinoma vs. squamous cell carcinoma) could not be distinguished in this cohort, preventing subtype-specific analysis. Fifth, due to the observational nature of the study, causal inferences cannot be made. Although significant associations were identified, these results do not establish direct biological effects. Sixth, although the primary aim of this study was to assess antioxidant responses under smoking-induced oxidative stress, the number of never-smokers was relatively small. Therefore, the lack of statistically significant associations in this subgroup should be interpreted with caution due to the potential for reduced statistical power. Lastly, although this study focused on male participants, the original cohort also included women. However, the number of smokers among women was substantially low, making it difficult to evaluate antioxidant responses related to smoking with sufficient statistical power. As such, women were excluded from the primary analysis to maintain consistency in the study design and ensure reliable interpretation of the results.

## 5. Conclusions

This study evaluated the associations between serum creatinine and representative endogenous antioxidant biomarkers such as bilirubin, albumin, and uric acid and the risk of lung cancer, stratified by smoking status. Creatinine showed a statistically significant inverse association with lung cancer risk, particularly among smokers, whereas no significant associations were observed with other major cancer types aside from lung cancer. These findings suggest that in smoking-related oxidative stress environments, endogenous antioxidant biomarkers including creatinine may have potential utility in predicting lung cancer risk. Further studies are warranted to clarify the underlying physiological mechanisms and the role of these biomarkers in cancer development.

## Figures and Tables

**Table 1:** Baseline characteristics of Korean cancer prevention study-II participants *.

Characteristics	Men (*n* = 83,371)	Women (*n* = 50,225)
Age, y	41.6 (9.5)	39.7 (10.7)
Body mass index ^†^	24.4 (2.9)	22.0 (3.0)
Serum creatinine	1.09 (0.22)	0.83 (0.15)
Serum bilirubin, mg/dL		
Total	0.95 (0.38)	0.75 (0.30)
Indirect	0.59 (0.26)	0.47 (0.21)
Direct	0.36 (0.14)	0.29 (0.12)
Albumin, g/dL	4.58 (0.25)	4.45 (0.25)
Uric acid, mg/dL	6.15 (1.24)	4.29 (0.89)
Alcohol drinking, g/d	22.67 (29.45)	5.98 (13.77)
Smoking status, %		
Never	22.7	89.4
Previous	32.7	6.4
Current	44.6	4.2
Any alcohol use, %		
Never	5.8	30.8
Previous	7.9	16.5
Current	86.3	52.7

* Data are expressed as mean (SD) unless otherwise indicated. Participants with any of the following features at study entry were excluded: missing data on serum bilirubin level, existing cancer, and missing data on smoking status. ^†^ Body mass index was calculated as weight in kilograms divided by the square of height in meters.

**Table 2:** Hazard ratios for lung cancer risk per 1 SD increase in serum creatinine: stratified by smoking status in men.

Serum Biomarker	Smoking Status	Case (Median Follow-Up Time, IQR)	HR (95% CI) ^§^	*p*-Value
Creatinine	^†^ Never-smokers	57 (13.7, 13.3–14.4)	1.079 (0.946–1.231)	0.2543
	^‡^ Never-smokers	57 (13.7, 13.3–14.4)	1.080 (0.941–1.239)	0.2751
	^†^ Former smokers	196 (14.2, 13.4–14.7)	0.774 (0.620–0.967)	0.0239
	^‡^ Former smokers	196 (14.2, 13.4–14.7)	0.770 (0.617–0.961)	0.0209
	^†^ Ever-smokers *	496 (14.1, 13.4–14.6)	0.823 (0.716–0.945)	0.0057
	^‡^ Ever-smokers *	496 (14.1, 13.4–14.6)	0.800 (0.697–0.920)	0.0017
	^§^ All men	553 (14.1, 13.4–14.6)	0.902 (0.794–1.025)	0.1134
	^||^ All men	553 (14.1, 13.4–14.6)	0.882 (0.776–1.002)	0.0541

Abbreviations: CI, confidence interval; HR, hazard ratio. * Ever-smokers were defined as current or former smokers. Each category was analyzed using two Cox models: one adjusted for BMI and one excluding BMI. ^†^ The Cox proportional hazards model was adjusted for age, sex, alcohol use, body mass index, GOT, and GGT. ^‡^ The Cox proportional hazards model was adjusted for age, sex, alcohol use, GOT, and GGT (BMI excluded). ^§^ The Cox proportional hazards model was adjusted for age, sex, smoking status, alcohol use, body mass index, GOT, and GGT. ^||^ The Cox proportional hazards model was adjusted for age, sex, smoking status, alcohol use, GOT, and GGT (BMI excluded).

**Table 3:** Association of antioxidant biomarkers with lung cancer risk by smoking status in men: per 1 SD increase in bilirubin, albumin, and uric acid.

Serum Biomarker	Smoking Status	Case (Median Follow-Up Time, IQR)	HR (95% CI) ^§^	*p*-Value
Bilirubin	^†^ Never-smokers	57 (13.7, 13.3–14.4)	0.848 (0.641–1.121)	0.2465
	^†^ Former smokers	196 (14.2, 13.4–14.7)	0.826 (0.705–0.967)	0.0177
	^†^ Ever-smokers *	496 (14.1, 13.4–14.6)	0.785 (0.708–0.870)	<0.0001
	^§^ All men	553 (14.1, 13.4–14.6)	0.829 (0.753–0.913)	0.0001
Albumin	^†^ Never-smokers	57 (13.7, 13.3–14.4)	0.840 (0.652–1.082)	0.1769
	^†^ Former smokers	196 (14.2, 13.4–14.7)	0.996 (0.871–1.139)	0.9582
	^†^ Ever-smokers *	496 (14.1, 13.4–14.6)	0.878 (0.807–0.955)	0.0024
	^§^ All men	553 (14.1, 13.4–14.6)	0.893 (0.825–0.968)	0.0059
Uric acid	^†^ Never-smokers	57 (13.7, 13.3–14.4)	1.15 (0.862–1.533)	0.3431
	^†^ Former smokers	196 (14.2, 13.4–14.7)	0.832 (0.699–0.989)	0.0367
	^†^ Ever-smokers *	496 (14.1, 13.4–14.6)	0.847 (0.760–0.944)	0.0027
	^§^ All men	553 (14.1, 13.4–14.6)	0.885 (0.799–0.981)	0.0203

Abbreviations: CI, confidence interval; HR, hazard ratio. * Ever-smokers were defined as current or former smokers. ^†^ The Cox proportional hazards model was adjusted for age, sex, alcohol use, body mass index, GOT, and GGT. ^§^ The Cox proportional hazards model was adjusted for age, sex, smoking status, alcohol use, body mass index, GOT, and GGT.

**Table 4:** Association between serum creatinine and lung cancer risk by smoking status in men: A quartile-based and trend analysis.

Serum Biomarker	Smoking Status	Case (Median Follow-Up Time, IQR)	Q1 HR (95% CI) ^§^	Q2 HR (95% CI) ^§^	Q3 HR (95% CI) ^§^	Q4 HR (95% CI) ^§^	*p* Value for Trend
Creatinine	^†^ Never-smokers	57 (13.7, 13.3–14.4)	1	1.087 (0.469–2.518)	0.802 (0.324–1.987)	1.603 (0.737–3.487)	0.2139
	^‡^ Never-smokers	57 (13.7, 13.3–14.4)	1	1.069 (0.462–2.473)	0.777 (0.314–1.922)	1.531 (0.706–3.317)	0.2589
	^†^ Former smokers	196 (14.2, 13.4–14.7)	1	1.155 (0.780–1.709)	0.605 (0.386–0.948)	0.854 (0.568–1.286)	0.0871
	^‡^ Former smokers	196 (14.2, 13.4–14.7)	1	1.145 (0.774–1.694)	0.599 (0.383–0.939)	0.843 (0.561–1.268)	0.0766
	^†^ Ever-smokers *	496 (14.1, 13.4–14.6)	1	0.920 (0.723–1.171)	0.689 (0.531–0.893)	0.757 (0.588–0.975)	0.0063
	^‡^ Ever-smokers *	496 (14.1, 13.4–14.6)	1	0.902 (0.709–1.148)	0.669 (0.516–0.867)	0.723 (0.562–0.929)	0.0019
	^§^ All men	553 (14.1, 13.4–14.6)	1	0.957 (0.759–1.207)	0.737 (0.574–0.947)	0.883 (0.696–1.120)	0.1143
	^||^ All men	553 (14.1, 13.4–14.6)	1	0.943 (0.748–1.189)	0.720 (0561–0.925)	0.847 (0.669–1.074)	0.0548

Abbreviations: CI, confidence interval; HR, hazard ratio. * Ever-smokers were defined as current or former smokers. Each category was analyzed using two Cox models: one adjusted for BMI and one excluding BMI. ^†^ The Cox proportional hazards model was adjusted for age, sex, alcohol use, body mass index, GOT, and GGT. ^‡^ The Cox proportional hazards model was adjusted for age, sex, alcohol use, GOT, and GGT (BMI excluded). ^§^ The Cox proportional hazards model was adjusted for age, sex, smoking status, alcohol use, body mass index, GOT, and GGT. ^||^ The Cox proportional hazards model was adjusted for age, sex, smoking status, alcohol use, GOT, and GGT (BMI excluded).

**Table 5:** Associations of antioxidant biomarkers with lung cancer risk by smoking status in men: Quartile and trend analyses.

Serum Biomarker	Smoking Status	Case (Median Follow-Up Time, IQR)	Q1 HR (95% CI) ^§^	Q2 HR (95% CI) ^§^	Q3 HR (95% CI) ^§^	Q4 HR (95% CI) ^§^	*p* Value for Trend
Bilirubin	^†^ Never-smokers	57 (13.7, 13.3–14.4)	1	0.870 (0.418–1.810)	0.522 (0.238–1.146)	0.860 (0.412–1.798)	0.4602
	^†^ Former smokers	196 (14.2, 13.4–14.7)	1	0.998 (0.667–1.493)	0.846 (0.569–1.259)	0.776 (0.501–1.201)	0.1671
	^†^ Ever-smokers *	496 (14.1, 13.4–14.6)	1	0.937 (0.744–1.180)	0.720 (0.568–0.913)	0.579 (0.436–0.770)	<0.0001
	^§^ All men	553 (14.1, 13.4–14.6)	1	0.969 (0.777–1.207)	0.758 (0.603–0.952)	0.691 (0.530–0.900)	0.0009
Albumin	^†^ Never-smokers	57 (13.7, 13.3–14.4)	1	0.700 (0.314–1.564)	0.637 (0.328–1.238)	0.581 (0.276–1.222)	0.1056
	^†^ Former smokers	196 (14.2, 13.4–14.7)	1	1.088 (0.718–1.651)	0.899 (0.614–1.314)	1.114 (0.768–1.617)	0.8060
	^†^ Ever-smokers *	496 (14.1, 13.4–14.6)	1	0.947 (0.734–1.222)	0.796 (0.633–1.001)	0.778 (0.606–1.000)	0.0183
	^§^ All men	553 (14.1, 13.4–14.6)	1	0.956 (0.749–1.219)	0.806 (0.649–1.001)	0.812 (0.640–1.030)	0.0303
Uric acid	^†^ Never-smokers	57 (13.7, 13.3–14.4)	1	0.767 (0.357–1.646)	1.652 (0.828–3.297)	1.184 (0.535–2.617)	0.3016
	^†^ Former smokers	196 (14.2, 13.4–14.7)	1	0.824 (0.566–1.199)	0.754 (0.500–1.137)	0.606 (0.385–0.954)	0.0244
	^†^ Ever-smokers *	496 (14.1, 13.4–14.6)	1	0.870 (0.693–1.093)	0.645 (0.492–0.845)	0.716 (0.544–0.942)	0.0016
	^§^ All men	553 (14.1, 13.4–14.6)	1	0.862 (0.693–1.072)	0.729 (0.568–0.935)	0.775 (0.598–1.004)	0.0134

Abbreviations: CI, confidence interval; HR, hazard ratio. * Ever-smokers were defined as current or former smokers. ^†^ The Cox proportional hazards model was adjusted for age, sex, alcohol use, body mass index, GOT, and GGT. ^§^ The Cox proportional hazards model was adjusted for age, sex, smoking status, alcohol use, body mass index, GOT, and GGT.

## Data Availability

The datasets, including summary statistics and R code generated during the current study, are available from the corresponding author upon reasonable request. Due to privacy and ethical restrictions, raw data are not publicly available.
